# Nervousness*Address to Mothers’ Congress, San Antonio, Texas, December, 1912, by F. S. White, M. D., Superintendent Southwestern Insane Asylum, San Antonio, Texas.

**Published:** 1913-04

**Authors:** 


### Nervousness.*

At the outset I must confess that I undertook the preparation of this paper with much perplexity and with grave doubts as to my ability to present the subject in such a manner as to meet your expectations. It is very difficult to present a medical or semi-medical subject to a non-medical audience in an interesting and instructive way, for the principal reason that the non-medical audi-

*Address to Mothers' Congress, San Antonio, Texas, December, 1912, by F. S. White, M. D., Superintendent Southwestern Insane Asylum, San Antonio, Texas.

ence abhors technical terms which often are necessary to make one's meaning clear.

Nervousness may be defined as an overexcitability of the nervous system, with a tendency to fatigue, especially of the muscular system, characterized by great unrest, mental and physical. This is the prodromal stage of what may become neurasthenia, hysteria or epilepsy. What the termination may be depends largely on the inherent characteristics of the individual organism as bequeathed to it, which may be modified or accentuated by environmental influences. If these influences are continuously unfavorable, we shall see hypochondriasis or melancholia develop according to the subject with which the mind of the individual is occupied, or even the false sensations or imperative ideas which usher in some form of insanity.

Nervousness is a comparatively modern affliction—a penalty, I might say—for which the individual is usually not entirely responsible. It is a developmental condition resulting from an unstable organization transmitted by ancestors and often unwittingly cultivated and developed by the individual sufferer.

Heredity, that great force by virtue of which peculiarities are transmitted from ancestors to offspring, and in accordance with whose inviolable laws races are perpetuated and all the different species of animals continue to exist, should be more closely studied today than any other subject. Its study should be given an important place in the curriculum of every school in this broad land.

If it were obligatory upon the present generation to acquire a comprehensive knowledge of the laws of heredity, its potentialities and momentous possibilities, the next generation would show a marked improvement over the present one. Idiocy, imbecility, crime and insanity would show a marked decrease. Abnormal nervousness would give way to a normal nervousness, which is an indication of increased capacity for work and endurance.

The enormous increase of nervousness within the last quarter of a century is due to heredity and environment. I could easily occupy the time allotted to me in discussing heredity, but have already said enough to show you the far-reaching effects of its influence.

Next to heredity as a factor in the causation of nervousness is environment, by which I mean all the influences and surroundings which enter into and form a part of the life of the individual.

We Americans are the fastest moving people on earth. We have long since passed the days of leisurely moving along. Where formerly we walked, we now run; where formerly we drove horses, we now drive automobiles at a speed which is a menace to not only our own lives, but to everything and everybody that chances to cross our path; and in a few more years we will probably abandon the fast-moving automobile for the more speedy and dangerous aeroplane. The main object seems to be to get there, and get there quickly.

This mad, frantic rush through life is leaving our pathway strewn with wreck and ruin. None but the strongest who have accidentally come of a hardy ancestry can stand the strain. The weaker are falling by the wayside and are adding to the burdens of the strong, as is evidenced by the great hordes of criminals, weak-minded and insane who must be cared for and supported by those who do not break down.

There seems to be prevalent among the people a spirit of dissatisfaction and unrest which urges them on to exert every particle of strength, both mental and physical, to excel others.

Men are seeking political and financial supremacy at the expense often of honor and health. Women are demanding and requiring more elaborate hats, finer gowns and more expensive lingerie for which the men must pay. Card parties and entertainments are becoming more and more elaborate, and this taxes to the uttermost the strength and resources of the hostess until, instead of being a pleasure, it is too often a sore burden for a lady to entertain her friends. All these things, and many more, are adding to the great number of nervous people and making us almost, if not quite, a race of neurasthenics.

This nervous and reckless, mad rush for preferment among the grownups has extended to the children, and is playing sad havoc with young and growing minds and bodies. Too often men and women, in order to push on a little further and a little faster, endeavor to whip up their flagging energies by resort to drugs and liquors, and in so doing many fall out of the procession and become confirmed neurasthenics and finally find a lodgment in our sanitariums for nervous and mental diseases, or our hospitals for the insane.

Now, ladies, I have very briefly laid bare to you a condition which is not pleasant to contemplate and one that must fill every mother's heart with great apprehension for the future of the boys and girls. Basing my opinion upon my experience in treating and caring for the wrecks of these conditions, I do not believe that I have overdrawn the picture.

Could you see the once bright and happy girls and the promising young men who are forced to take advantage of a home prepared by the State, you would say that the half has not been told. Could you see the wan faces, emaciated bodies and tired eyes of the noble and self-sacrificing mothers who come under my care, you would agree with me that something is seriously wrong with the social fabric of this country. I tell you that too many defective people are being made.

I consider it a disgrace for parents to force into existence a child without endowing it with the potentialities of becoming strong and healthy. To do so in this enlightened age is an unpardonable sin against humanity. It were better never to be born than to be born to inevitably become a criminal or to become insane from inheritance.

The important question is how to remedy these conditions. Intelligent education and regulations of surroundings can do much to prevent the development of inherited weaknesses.

The child with a nervous ancestry should be carefully protected from influences which have a tendency to bring out such latent defects. First of all, the child should be made to grow by being supplied with an abundance of good, nourishing food. More attention should be given to the growth of the body than the mind. Plenty of sunshine and fresh air is as indispensable to the growth of a child as it is to the growth of a plant.

Such children should not be sent to school before the seventh year and should not then be encouraged to study hard. Their development and progress should be closely watched, and upon the first appearance of nervous symptoms they should be taken out of school and their books hidden and in their stead should be given a fishing pole, ball, hoop, marbles and a stick horse. Especial care should be given these unfortunate children at the approach of puberty. There are today many nervous girls and women who would be strong and healthy had they been taken out of school and away from study of every kind at this important crisis.

Every child who displays any symptoms of nervousness should at once have the services of a competent, broad-minded physician, who will carefully examine them from head to foot. It is not uncommon for mothers to disregard the advice of their physicians in regard to the raising and care of a nervous child, at the expense of the child's health and future happiness. Grades at school must be made.

The future hope of this country rests with the mothers. You who have read "Keeping Up With Lizzie" will recall that Socrates Potter said of Mrs. Warburton: "She has discovered that there are only three luxuries—work, children and motherhood; that to shift responsibility is to forfeit happiness."

When the mothers of this country discover these great truths, the millennium will have come and in a short time nervousness will be a thing of the past.

As I see the conditions, the greatest good you can accomplish is to teach these great truths to the mothers of the land. I believe that the grandest heritage that any woman can leave on earth is a family of healthy sons and daughters, who will be a solace and comfort to her declining days and an honor to her generation, and I believe further that any woman who fails to become a mother has failed in the accomplishment of the object for which she was created.

Now, ladies, I may have expressed my opinions too plainly to meet your approbation, but, after practicing medicine for over a quarter of a century, I do not know how to speak otherwise.

I am most happy to avail myself of this opportunity to commend you noble mothers for the great work you are engaged in. Your work is the improvement and ennobling of the human race, than which there is no greater, and I indulge the hope that results commensurate with your ideals will crown your efforts.

Oliver Wendell Holmes, in his beautiful tribute to motherhood, says: "The woman about to become a mother, or with the newborn infant upon her bosom, should be the object of trembling care and sympathy wherever she bears her tender burden or stretches her aching limbs. The very outcast of the streets has pity upon her sister in degradation when the seal of promised maternity is impressed upon her. The remorseless vengeance of the law, brought down upon its victim by a machinery as sure as destiny, is arrested in its fall at a word which reveals her transient claim for mercy. The solemn prayer of the liturgy singles out her sorrows from the multiplied trials of life, to plead for her in the hour of peril."

---

When there is persistent irritation of the throat without local cause, examine the chest. This may be one of the earliest symptoms of mediastinal tumor or enlarged bronchial glands.—*American Journal of Surgery.*

---

If one end of a needle projects superficially, by squeezing the muscles in the proper direction, from beneath its deeper end, it can often be driven through the skin and extracted without incision.—*American Journal of Surgery.*

